# Supplementary figures and images for: Demographic analysis of an Israeli Carpobrotus population
Source: PLoS One. 2021 Apr 30;16(4):e0250879. doi: 10.1371/journal.pone.0250879 (PMC8087044; doi:10.1371/journal.pone.0250879)

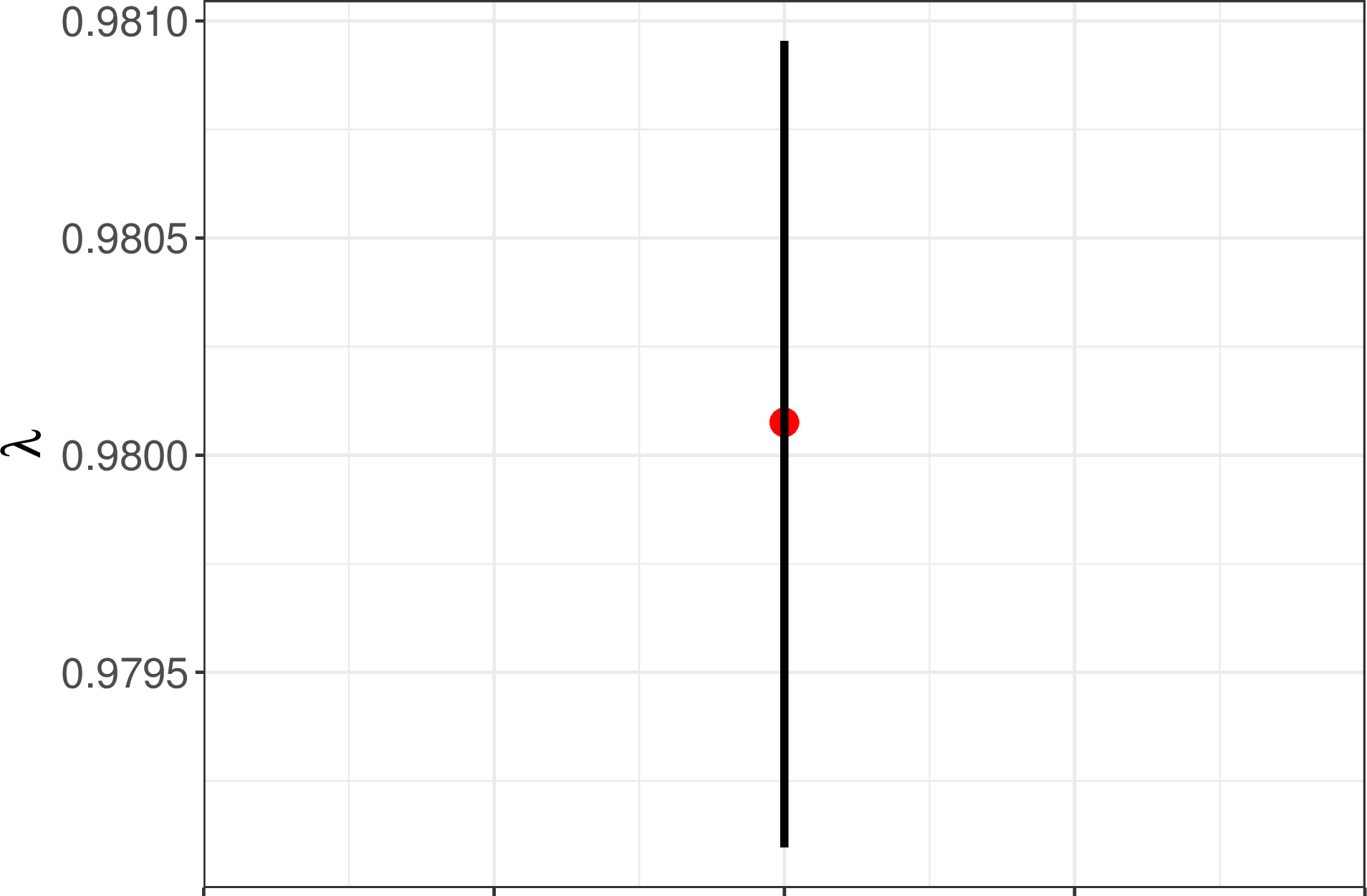

Supplement: S1 Fig — The red point is the per-capita growth rate estimated from the regression parameters. The black line shows the upper and lower 95% confidence intervals derived from the draws that perturbed each flower number regression parameter. The range of values is quite small, and our qualitative results are not affected by the assumption of little temporal variation in flower production. (TIF) [file pone.0250879.s001.tif]
