# Supplementary material for: Demographic analysis of an Israeli Carpobrotus population
Source: PLoS One. 2021 Apr 30;16(4):e0250879. doi: 10.1371/journal.pone.0250879 (PMC8087044; doi:10.1371/journal.pone.0250879)
Supplement: S1 File — S1.1 Table. Statistical values for the vital rates parameters. Coefficients, standard errors, test statistics, and p-values for each of the vital rate regression used in the IPM to generate point estimates of lambda, sensitivity, and elasticity functions. S1.2 Table. AIC model selection tables for candidate growth models. S1.3 Table. AIC model selection tables for candidate survival models. S1.4 Table. AIC model selection tables for candidate probability of flowering models. S1.5 Table. AIC model selection tables for candidate flower production models. (PDF) [file pone.0250879.s002.pdf]

IPM Functions

1. $n(z', t+1) = \int_L^U K(z', z)n(z, t)dz$
2. $K(z', z) = P(z', z) + F(z', z)$
3. $P(z', z) = s(z) * g(z', z)$
4. $F(z', z) = p_r(z) * r_s(z) * r_d(z') * r_r$

NB: below, f_g and f_{r_d} denote truncated Gaussian probability density functions (with truncation interval $[L, U]$).

P(z',z)

5. $Logit(s(z)) = \beta_{0,s} + \beta_{1,s} * z$
6. $g(z', z) = f_g(z', \mu_g, \sigma_g)$
7. $\mu_g = \beta_{0,g} + \beta_{1,g} * z$
8. $\sigma_g = \sqrt{e^{2*\beta_{\sigma_g}*z}}$

F(z', z)

9. $Logit(p_r(z)) = \beta_{0,p_r} + \beta_{1,p_r} * z$
10. $ln(r_s(z)) = \beta_{0,r_s} + \beta_{1,r_s} * z$
11. $r_d(z') = f_{r_d}(z', \mu_{r_d}, \sigma_{r_d})$

Implementation rules

Integration rule: midpoint

L = -7.256

U = 3.979

meshpoints = 100

Drone results

The drone surveys from 2018 required 6 batteries over the course of 2 days due to a limited battery supply. Ambient temperature was approximately 24 degrees and there were no clouds present during either sampling period. In total, 1720 images were taken of the population from a height of ~9m above ground level. Due to difficulties in following the terrain perfectly, pixel sizes varied, though were on average 0.26cm/pixel. Pix4DMapper successfully geolocated and matched 1695/1720 images across 6 blocks, with a median of 10005.9 matches/image and a 1.83% difference in initial and optimized camera parameters.

The 2019 survey was conducted on a single day under similar conditions to 2018. 1024 images were collected 10-15m above ground level to increase the variability within each image and prevent Pix4DMapper from generating blocks during orthomosaic generation. 1021/1024 images were successfully calibrated in a single block with a mean resolution of 0.45cm/pixel. Despite the drop in resolution, flowers and plants were still quite easy to identify in the resulting orthomosaic. Images had a median of 10219.3 matches, and there was a 0.35% difference between initial and optimized camera parameters.

At each sampling, squares of known size were also recored in each image and had polygons drawn around them (each target measured 0.375m X 0.315m). Thus, we were able to compute the ratio of observed surface areas to true surface areas for the orthomosaic. Observed plant surface areas were standardized using the ratio of the true size to the observed sizes.

Model fitting results

Table S1.1: Coefficients, standard errors, test statistics, and p-values for each of the vital rate regression used in the IPM to generate point estimates of lambda, sensitivity, and elasticity functions.

Vital Rate	Parameter	Estimate	Std. Error	Test Statistic	P-value
Growth	Intercept	0.02862	0.0397	0.721	4.72e-01
	Slope	0.89849	0.0252	35.595	9.59e-96
	Sigma Exponent	-0.1754			
Survival	Intercept	2.6963	0.313	8.60	7.84e-18
	Slope	0.7312	0.144	5.09	3.51e-07
Pr(Flowering)	Intercept	0.8801	0.192	4.59	4.33e-06
	Slope	1.1028	0.136	8.09	6.20e-16
Flower Production	Intercept	1.568	0.0453	34.7	3.47e-263
	Slope	0.753	0.0238	31.6	1.07e-219
Recruit Size	Mu	-2.975			
	Sigma	1.064			

Table S1.2: AIC model selection tables for candidate growth models.

Model	Type	df	AIC
Growth	Intercept Only	2	863.1472
Growth	Linear Term + Intercept	3	483.1242
Growth	Quadratic Term + Intercept	4	484.9146
Growth	GAM	3	483.1242
Growth	Exponential Variance	4	459.3743

Table S1.3: AIC model selection tables for candidate survival models.

Model	Type	df	AIC
Survival	Intercept Only	1.000	240.8141
Survival	Linear Term + Intercept	2.000	208.5736
Survival	Quadratic Term + Intercept	3.000	209.2357
Survival	GAM	2.282	208.5197

Table S1.4: AIC model selection tables for candidate probability of flowering models.

Model	Type	df	AIC
Probability of Reproduction	Intercept Only	1	384.3853
Probability of Reproduction	Linear Term + Intercept	2	273.8571

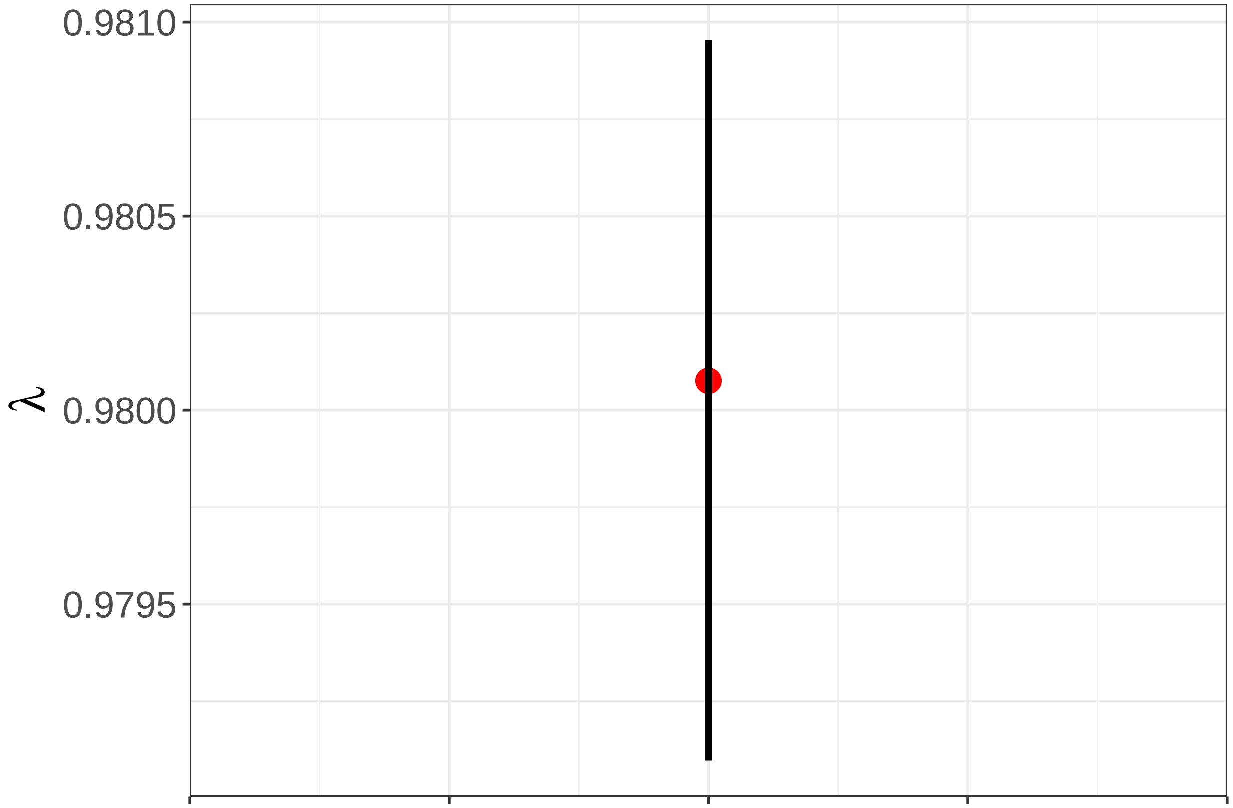

Figure 1: Results for the flower number regression parameter perturbation analysis. The red point is the per-capita growth rate estimated from the regression parameters. The black line shows the upper and lower 95% confidence intervals derived from the draws that perturbed each flower number regression parameter. The range of values is quite small, and our qualitative results are not affected by the assumption of little temporal variation in flower production.

Table S1.5: AIC model selection tables for candidate flower production models.

Model	Type	df	AIC
Flower Production	Intercept Only	1	2039.4341
Flower Production	Linear Term + Intercept	2	890.0934
