# Supplementary material for: Demographic analysis of an Israeli Carpobrotus population
Source: PLoS One. 2021 Apr 30;16(4):e0250879. doi: 10.1371/journal.pone.0250879 (PMC8087044; doi:10.1371/journal.pone.0250879)
Supplement: Attachment — Submitted filename: 21 02 17 Revision Mfos.pdf [file pone.0250879.s003.pdf]

General comments

I revised the manuscript entitled "Demographic analysis of Israeli Carpobrotus populations: management strategies and future directions".

I found some merits in the manuscript which deals an interesting topic, providing demographic data using drones. Nevertheless, the whole paper should be extensively revised and rewritten.

Title:

The title should be revised and focused on the type of research, the use of images obtained by drone. The study has only been conducted in one population. "Management strategies and future directions" do not seem justified to include it in the title.

Abstract:

Line 22 (L22) delete (UAVs).

L29 – *Aizoaceae* italics.

L31 – confirm by demonstrate.

Introduction

The introduction should be restructured. Information is there but is pretty messy.

Invasive species and the importance of coastal ecosystems (L66-72) could be reduced and could join with the problem of invasive species (L35-36).

The description of Carpobrotus (L37-43), its impacts (L44-51) and the investigations (L56-59) should be rewritten and joined in a paragraph. Research on eradication and the effects of control actions should be mentioned. See below recent papers:

Chenot et al. (2018) Restor Ecol 26:106-113.

Lazzaro et al. (2020) Biologia 75:199-208.

Buisson et al. (2020), Applied Vegetation ScienceVolume 24, Issue 1.

Braschi et al. (2021) Biodiversity and Conservation (2021) 30:497–518.

Fos et al. (2021) Plant Biosystems.

The part dedicated to "Structured population models" in Carpobrotus (L 52-59, L 62- 65) and "Structured population models" in dune ecosystems (L72-79) should be shortened and joined in one paragraph.

L37 - *Carpobrotus* N. Br. (*Aizoaceae*).

L38 – sensitive by frail.

L40 – Correct botanic names *C. edulis* and *C acinaciformis.*

L50 – Allelopathic properties of the *Carpobrotus* litter also inhibit emergence (Fos et al., 2021.

L55 – can be rewritten: "management, managers, management" in the same sentence.

L59-62 – "Succulents are an .... and Opuntia spp (29)" delete the paragraph, the information is not relevant.

L86-87 delete “unmanned aerial vehicles” and “Integral Projection Models”.

L92 – confirm by demonstrate.

Methods

The methods are written in a single section and they must organize in specific sections.

Fig. 1 - include scale.

L112-119 reduce paragraph, relevant information is only L115-116.

The use of the terms “plant” and “ramet” is confusing.

L160 4 m instead of 4m.

L190 delete “Integral Projection Model”.

Results

The results should include descriptive information from the experimental data, for example: total area occupied by *Carpobrotus,* mean plant area, maximum and minimum plant area, frequency by size of plant area, number of flowers and fruits per plant, etc. and variations from 2018 to 2019.

L 235 why C. edulis ???.

Discussion

The discussion should be revised, shortened and rewritten.

Include table and figure references in the discussion.

L273 – *Aizoaceae* italics.

L279 - delete “Integral Projection Models”.

L269-275 - This paragraph is better included in the introduction.

L282-284 - Delete “Individual ...... vertical cliff”.

L289-291 “Third, it is ... for Carpobrotus” - this sentence is contradictory with the references on invasive behavior and growth of Carpobrotus, (see Campoy et al 2018),

L297-298 - No data is presented regarding it, see Result comments.

L303-329 - This part should be rewritten by joining the 2 paragraphs. Recent eradication works and the effects of control actions should be included and discussed. See papers in introduction comments. It would be interesting to discuss the contributions of this work and the potential of the use of drones at least in the monitoring of regenerated areas.

L305-306 – “It is ... growth” too speculative.

L310-311 - In the case of Carpobroptus, the actions should be aimed at the complete eradication and not at the reduction of the population.

L330-338 - This part is very general and it has nothing to do with the work they present.
